# Consciousness and spintronic coherence in microtubules

**DOI:** 10.1080/19420889.2025.2576334

**Published:** 2025-10-21

**Authors:** Majid Beshkar

**Affiliations:** Tehran University of Medical Sciences, Tehran, Iran

**Keywords:** Axon initial segment, consciousness, microtubule

## Abstract

Microtubules are nanoscale spintronic oscillators with memristive properties. Spintronic and memristive effects, together with some unique conditions found in the axon initial segment (AIS), allow quantum coherence to emerge spontaneously in a population of microtubules located within the AIS. According to the QBIT theory, the spontaneous emergence of coherence in a population of microtubules is the necessary and sufficient condition for the generation of a micro-consciousness (a quale) by the brain. Simultaneous generation of multiple qualia by synchronized activity in different parts of the cerebral cortex gives rise to the appearance of a macro-consciousness: a seemingly unified subjective experience.

## Introduction

According to the QBIT theory of consciousness, qualia (plural for quale) are the building blocks of consciousness. Each quale (also called a micro-consciousness) is generated by a specific part of the cerebral cortex. The seemingly unified subjective experience that an individual has at any given moment (called macro-consciousness) is composed of multiple distinct qualia generated simultaneously by different cortical areas. In this context, the relationship of each micro-consciousness (quale) to the overall macro-consciousness can be likened to the relationship between the pixels on a television screen and the overall picture that is present on that screen at a given moment. This idea (which can be called the disunity of consciousness) is strongly supported by experimental findings in the visual system (for a review, see [1]).

From the perspective of the QBIT theory, the generation of a micro-consciousness depends on a specific substrate, a suitable environment, and a particular mechanism. The substrate is the microtubule, the environment is the axon initial segment, and the mechanism is the spontaneous emergence of coherence [2].

## Microtubules

In digital computers, the basic unit of computation is the transistor [3]. What is the basic computational unit of the brain? According to the QBIT theory, the answer is the microtubule. In other words, the microtubule is the main substrate with which the brain performs a variety of different computations and information processing tasks, including those involved in sensory perception and consciousness.

Interestingly, a look at the electrical properties of microtubules gives the impression that they are emulating neurons. In other words, each microtubule plays the role of a neuron for the cytoplasmic environment: a nerve inside the cell [4]. Similar to neurons, microtubules are highly sensitive to electrical stimulation, behaving as nonlinear transmission lines capable of amplifying and axially transferring electrical signals [5]. Even in the absence of external stimulation, microtubules spontaneously generate electrical oscillations and bursts of electrical activity similar to action potentials produced by neurons [6].

The idea that microtubules emulate neurons and serve as the basic computational units of the brain receives further support from emerging evidence indicating that microtubules have memristive properties. Cantero et al. [7], Tuszynski et al. [8], Kalra et al. [9], and Gutierrez et al. [10] have highlighted the memristive properties of microtubules, suggesting that microtubules actually operate as nanoscale memristors inside the cell.

A memristor (a resistor with memory) is an electronic device initially hypothesized by Chua [11], and experimentally realized for the first time by Strukov et al. [12] utilizing titanium dioxide as a substrate. Since then, various types of memristors have been fabricated using different organic and inorganic substrates [13].

Memristors can store and process information [14]. When incorporated within an appropriate circuit, memristors can perform logic operations [15]. It is important to note that any form of computation (whether classical or quantum) can be decomposed into elementary logic operations [16].

Memristors spike naturally in a manner qualitatively similar to neurons [17]. In general, with their working mechanisms based on ion movement, the electrical behavior and switching dynamics of memristors closely resemble those of synapses and neurons [18]. This resemblance makes memristive devices exceptionally promising candidates for neuromorphic (i.e., brain-like) computing.

Pershin and Di Ventra [19] have demonstrated that a memristor can function as a neuronal synapse, and when memristors are combined together in networks, they reproduce an important function of the brain, namely the associative memory. Interestingly, a network of memristors can solve complex mazes [20], a functional property that implies (at least) a primitive form of intelligence and bears resemblance to the cognitive abilities of the brain.

Several research groups have successfully fabricated artificial sensory neurons using memristive devices. For example, Kim et al. [21] have developed a memristor that reproduces the functions of a nociceptive neuron by exhibiting four specific nociceptive behaviors (i.e., threshold, relaxation, allodynia, and hyperalgesia) according to the strength, duration, and repetition rate of external stimuli. Similarly, Wen et al. [22] have utilized memristors to fabricate artificial visual neurons that exhibit sensory and oscillatory behaviors typical of biological neurons. Yang et al. [23] have demonstrated that memristive oscillators can perform sophisticated sensory processing tasks such as touch recognition and gesture recognition. In general, memristive oscillators have the remarkable ability to integrate sensing, storage, and computation by dynamically modifying their intrinsic frequencies in response to sensory stimuli. In this framework, information is encoded and transmitted by means of phase differences [23]. Given that memristors are capable of mimicking the sensory and computational functions of neurons, and considering that microtubules exhibit memristive characteristics, it may be argued that the sensory and computational capacities of neurons originate from, and are fundamentally driven by, the activities of microtubules.

According to the QBIT theory, microtubules not only possess memristive characteristics but also exhibit spintronic properties, and behave as spintronic oscillators. Spintronics (an abbreviation of spin electronics) fundamentally differs from conventional electronics in that, it uses the spin (rather than the electric charge) to encode and process information [24]. In this context, the term “spin” refers to the intrinsic angular momentum of a charged particle (the electron or the nucleus). A spin could be regarded as a tiny magnet that exists in either an “up” or a “down” state.

While the memristive properties of microtubules are substantiated by a growing body of evidence, the idea that microtubules exhibit spintronic effects remains largely speculative, with only a limited amount of indirect evidence to support it. Despite the lack of sufficient evidence, the QBIT theory insists that microtubules must possess spintronic properties, and predicts that these properties will be revealed in future experiments. This insistence is based on the fact that quantum coherence (which is a necessary condition for the emergence of consciousness) is more stable at physiological temperatures when it is spintronic rather than electronic or vibrational in nature. The QBIT theory suggests that, in the warm and noisy environment of the brain, microtubules can remain in a coherent state for a sufficiently long time only if they exhibit spintronic properties.

Manipulation of spins is essential for spintronic computation and information processing. In general, spins are manipulated in one of the two ways: with an external magnetic field, or through the spin – orbit interaction, which is stronger for heavier atoms such as ytterbium [24]. Therefore, one would not expect a substrate that is neither magnetic nor composed of heavy atoms to be relevant to spintronics at all. Microtubules are not magnetic, and they are mainly composed of light atoms such as carbon, hydrogen, and oxygen. As a consequence, it is not reasonable to assume that microtubules exhibit spintronic effects. Prior to the discovery of spintronic effects in deoxyribonucleic acid (DNA) by Göhler et al. [25], it was widely believed that such effects were unlikely to occur in molecules composed of light atoms, including DNA. However, findings in DNA spintronics have clearly demonstrated that spintronic phenomena can indeed manifest in molecular systems that are based on light atoms.

Similar to the microtubule, DNA is neither magnetic nor composed of heavy atoms. However, experimental evidence clearly demonstrates that, under specific conditions, DNA can actually exhibit strong spintronic effects at room temperature [25]. DNA molecules exhibit spintronic properties only when they are arranged as a closely packed and ordered collection of helices. Furthermore, the longer the helices, the stronger the spintronic effects. By contrast, a disordered population of loosely arranged DNA molecules fails to exhibit any spintronic properties [26].

The unexpected spintronic nature of DNA revealed by Göhler et al. [25] is mainly related to the structural properties of DNA, particularly the fact that DNA is a chiral molecule [24]. Furthermore, the finding that only a well-ordered and compact population of long DNA molecules can exhibit spintronic effects indicates that the way chiral molecules are assembled together determines whether or not the assembly can exhibit spintronic properties [26]. It is noteworthy that, like DNA, microtubules are also chiral molecules [27]. Furthermore, in the axon initial segment, relatively long microtubules assemble into well-ordered bundles in which microtubules are kept in close proximity to each other. This hints at the possibility that microtubules might exhibit spintronic effects within the axon initial segment.

In general, spin degrees of freedom have longer coherence times than charge degrees of freedom because the coupling of the environment to spin is weaker than to charge [28,29]. Therefore, decoherence (i.e., the loss of quantum coherence as a result of the coupling to the environment) could occur at a slower rate in a molecule with spintronic properties compared to a molecule that exhibits only conventional electronic properties. Electronic coherences typically decay in less than 1 picosecond, vibrational coherences persist for several picoseconds, while electron and nuclear spin coherences have significantly longer lifetimes, ranging from microseconds to milliseconds and milliseconds to seconds, respectively [30]. Spintronic coherence can persist for relatively long times even at room temperature, and this has been demonstrated for several substrates including diamond with a life time of 1.8 milliseconds [31,32], and a chromophore – radical molecular system with a lifetime of 0.7 microseconds [33].

One of the most promising substrates for spintronic computation is the carbon nanotube, a non-magnetic material in which spintronic coherence can persist for exceptionally long periods of time, up to 10 seconds and even more [34]. In carbon nanotubes, spins can be manipulated solely through electrical signals, for example an oscillating electric field [35,36]. This implies that, using carbon nanotubes, it is possible to transform electrical signals into information encoded on spins. Furthermore, it has been demonstrated that by exploiting the spintronic properties of carbon nanotubes, it is possible to transform information encoded on spins into a large electrical signal [37]. These findings collectively suggest that carbon nanotubes are capable of receiving electrical signals, converting them into information encoded on spins, and subsequently transforming this information into another electrical signal.

Carbon nanotubes represent one of the most promising products of nanotechnology, and microtubules are one of the most fascinating products of the evolution of life. In many respects, carbon nanotubes are regarded as the closest technological counterpart of microtubules [38]. The striking resemblance between carbon nanotubes and microtubules provides a basis, although tenuous, to suggest that, similar to carbon nanotubes, microtubules might also be capable of exhibiting spintronic properties. From this perspective, microtubules can be regarded as spintronic computers that receive electrical signals (from the cell membrane or the intracellular environment), convert the signals into spin-based information, perform spintronic computations on that information, and finally convert the information back into electrical signals.

Spintronic oscillators are very promising as the substrate for neuromorphic computing [39]. Spin-based neuromorphic chips could potentially be more compact and energy efficient compared to their electronic counterparts [40]. Even a single nanoscale spintronic oscillator can perform impressive cognitive computations. Torrejon et al. [41] have experimentally demonstrated that a single nanoscale spintronic oscillator can emulate a full neural network consisting of 400 neurons and perform speech recognition tasks with an accuracy of 99.6%.

In addition to compactness and low power consumption, spintronic oscillators have an exceptional capacity to synchronize their rhythms not only with adjacent spintronic oscillators but also with an oscillating electrical signal that serve as an input. This extraordinary capacity allows spintronic oscillators to learn from inputs and subsequently to differentiate between various external stimuli. In this context, learning is achieved by adjusting the frequencies of spintronic oscillators such that different patterns of synchronization among the oscillators are associated with different groups of input signals. Romera et al. [42] have demonstrated that, by exploiting the ability of spintronic oscillators to adjust their frequency in response to injected electrical currents, it is possible to train a network of four spintronic oscillators to categorize different input patterns into different synchronization configurations. The authors have shown that such a simple network can successfully learn to perform impressive cognitive tasks, such as distinguishing between vowels pronounced by different speakers.

The learning capacities of spintronic oscillators can be further enhanced by adding memristive properties to the system. Research indicates that incorporation of memristors into a network of nanoscale spintronic oscillators allows for the control of oscillation frequencies and the emergence of synchronization patterns [43]. This will enable the oscillators to learn through association similar to neurons in the brain [44].

In summary, there are numerous reasons to assume that microtubules are nanoscale spintronic oscillators with memristive properties, and that these spintronic and memristive properties allow microtubules to perform neuromorphic computations inside the cell. Microtubules are the main substrate with which the brain computes. However, it should be emphasized that neuroscience has effectively explained neural dynamics in terms of action potentials and synaptic transmission, which form the basis of behavior. The QBIT theory’s assertion that microtubules serve as the primary substrate by which the brain computes does not aim to refute the well-established role of action potentials and synaptic computations in brain dynamics. Rather, this proposal seeks to highlight the potential that electronic and spintronic oscillations of microtubules may underlie or modulate spiking activities of individual neurons and neuronal populations. This idea is in line with the findings of a patch clamping study conducted by Gutierrez et al. [45], which provides direct evidence that the honeybee brain produces intrinsic electrical oscillations that are mediated by intracellular microtubules. On the basis of these findings, Gutierrez et al. [45] have suggested that microtubules serve as the brain central oscillator that underlie all the electric waves observed in the brain.

If microtubules are conceptualized as nanoscale spintronic oscillators through which the brain performs computational (or information processing) functions, two fundamental questions arise: first, by what mechanism is information encoded within microtubules; and second, how do the spintronic oscillations of microtubules influence the membrane potential and consequently modulate neuronal firing? These questions present significant challenges for which the QBIT theory currently lacks evidence-based responses. However, preliminary hypotheses can be proposed: firstly, information may be encoded in the amplitude, phase, or a combination of both parameters of the spintronic oscillations within microtubules. Secondly, given that spintronic oscillators generate oscillating voltages with variable amplitude and frequency in response to direct current [46], it is plausible that such voltages, if propagated from the microtubule surface to the neuronal membrane, could influence the membrane potential and thereby modulate neuronal firing.

## Axon initial segment

The axon initial segment (AIS) is a unique neuronal compartment that is located immediately after the axon hillock, extending along the first 20–50 micrometers of the axon [47]. The AIS serves as the spike trigger zone, a site for the final integration of synaptic inputs and the initiation of action potentials. Compared to other neuronal compartments, the AIS has the lowest threshold for spike generation, primarily because it has an exceptionally high density of voltage-gated sodium channels. Furthermore, the AIS has other voltage-gated ion channels that are particularly sensitive to relatively minor deviations from the resting membrane potential. All these channels together play a critical role in transforming graded synaptic potentials into a train of action potentials [48, p. 231]. Therefore, the AIS electrically connects the somatodendritic compartment to the rest of the axon, integrating all the synaptic inputs and converting them into a temporally precise action potential code [49]. In a sense, the AIS is the site where the neuron decides whether to spike or not to spike.

A characteristic feature of the AIS is that it contains a unique protein scaffold located under the plasma membrane, spanning from the membrane to microtubules. This scaffold keeps microtubules in close proximity to the membrane, at a distance of approximately 50 to 100 nanometers [50]. The central component of this scaffold is ankyrin G, a protein that is observed exclusively in the AIS and nodes of Ranvier [51]. Ankyrin G contains a membrane-binding domain on one side and a microtubule-binding domain on the other side. The microtubule-binding domain connects to microtubules via linker proteins such as EB1, EB3, and Ndel1 [52].

The AIS microtubules have several characteristics that distinguish them from those located in other parts of the neuron. In the AIS, single or isolated microtubules are rarely observed [53]; instead, microtubules are linked together, forming tight bundles of 3 to 10 closely apposed parallel microtubules known as fascicles [50]. In dendrites and the soma, microtubules are not fasciculated. Although fasciculation of microtubules starts at the axon hillock, most of the microtubules in the axon hillock are non-fasciculated [54]. The fasciculation of microtubules ceases abruptly at the end of the AIS and the beginning of the myelin sheath [53]. Unlike the AIS (where microtubules are densely packed into fascicles), at the distal parts of the axon, microtubules are uniformly dispersed within the axoplasm [55].

In general, microtubules located in the AIS are more stable that those located in dendrites and distal parts of the axon [55,56]. The AIS microtubules are enriched in post-translational modifications (such as acetylation, detyrosination, and polyglutamylation) that make microtubules particularly stable [57].

**Figure 1.**
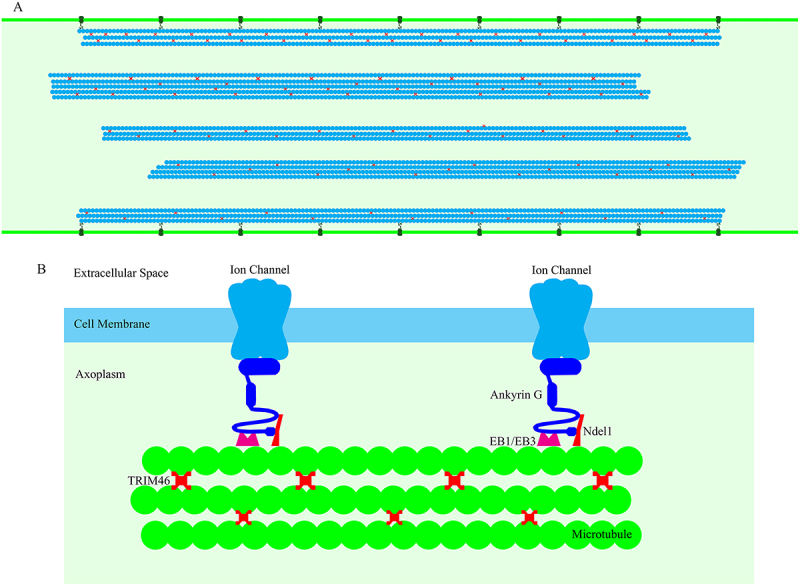
Schematic illustration of the axon initial segment (AIS). A: In the AIS, some microtubule fascicles are kept in close proximity to the cell membrane, while others are located further away from the membrane within the axoplasm. B: ankyrin G is essential for keeping microtubules close to the cell membrane. On the one side, ankyrin G binds to the transmembrane ion channel, and on the other side, it connects to the microtubule via EB1/EB3 and Ndel1. TRIM46 plays an important role in the assembly of microtubule fascicles by cross-linking the microtubules.

In all parts of the axon, including the AIS, microtubules have uniform orientation, with the minus-ends pointing toward the cell body and the plus-ends pointing toward the tip of the axon. By contrast, dendritic microtubules have mixed orientation, with their plus ends facing either the cell body or the tip of the dendrite [58]. TRIM46 (a microtubule cross-linking factor) contributes to the formation of closely spaced parallel microtubule bundles in the proximal axon, and plays a major role in the uniform orientation of microtubules in the AIS [59]. Furthermore, TRIM46 acts as a rescue factor, leading to the formation of microtubules that are stable and long [60]. TRIM46 is absent in the cell body and dendrites but highly enriched in the initial part of the axon [58]. Figure 1 provides a schematic representation of the AIS, highlighting some of its essential components including the fascicles, Ankyrin G, and TRIM46.

According to the QBIT theory, the characteristic features of the AIS allows microtubules to exhibit spintronic effects, and facilitates the emergence of long-lived quantum coherence in the AIS microtubules. As mentioned in the previous section, by extrapolating from the experimental findings about the spintronic properties of DNA [25], the QBIT theory suggests that the highly organized structure of a microtubule fascicle is a necessary condition for microtubules to exhibit spintronic effects. This high level of organization is reflected in the presence of closely apposed, long, stable, parallel, and uniformly-oriented microtubules. This condition is not present in dendrites where microtubules have mixed orientation, representing a form of disorganization.

The close opposition of the AIS microtubules is probably an important factor in the emergence of a coherent collective mode of activity, because research shows that the distance between nanoscale spintronic oscillators significantly affects their ability to synchronize (i.e., to phase lock with each other). For example, Kaka et al. [61] have demonstrated that two nanoscale spintronic oscillators can achieve phase locking when they are separated by 500 nanometers (nm), but not when they are separated by 1000 nm. Similarly, Mancoff et al. [62] have demonstrated that two 80-nm-diameter nanoscale spintronic oscillators will phase-lock to each other (with zero phase shift) when the spacing between them is less than 200 nm.

Research on the second-harmonic (SH) generation by microtubules provides some rudimentary evidence implying that uniform and parallel organization of microtubules is essential for the emergence of quantum coherence in microtubules [63, p. 21]. SH generation is a nonlinear quantum optics phenomenon that is related to the coherent activity of a system’s components. The generation of an SH signal by a system strongly depends on the efficient constructive interference among its components [64].

SH generation is a rare phenomenon in biology, occurring only in asymmetric, optically nonlinear structures that are hyperpolarizable. Only a few biological systems (including collagen and microtubules) can generate SH signals [65]. Microtubules exhibit this phenomenon because tubulin dimer is hyperpolarizable and asymmetric along its longitudinal axis.

Although SH generation is a universal property of all microtubules, axonal microtubules are more efficient in generating SH signals compared to microtubules in dendrites and soma [66]. This is partly due to the uniform orientation of axonal microtubules which results in the SH signals from individual microtubules to be parallel-aligned. This phase matching among signals leads to constructive interference and amplification of the overall signal. Conversely, dendritic microtubules have mixed orientations which leads to partial destructive interference due to phase mismatching. This may cause the intensity of the overall signal to fall below detectable levels, or in instances of complete symmetry, effectively reduce to zero [64].

In addition to the uniform orientation, the distance between microtubules is also a critical factor in SH generation by microtubules [63, p. 22]. Compared to a bundle of closely opposed microtubules, a population of dispersed microtubules is less likely to generate a significant SH signal. Furthermore, the number of microtubules, their stability, and the amount of order in a population of microtubules also contribute to the capacity of the population to generate SH signals [64]. The more the number of microtubules, and the higher the level of organization, the greater the intensity of the signal. Van Steenbergen et al. [64] have demonstrated that SH signals generated by microtubules mainly originate from stable GTP-bound tubulin dimers, due to their larger dipolar hyperpolarizability.

In summary, based on some indirect evidence, the QBIT theory suggests that the unique position of the AIS within the neuron, together with the characteristic features of AIS microtubules, create an optimal setting for the emergence of long-lasting quantum coherence in microtubules. No other part of the neuron can support the generation and maintenance of spontaneous coherence in microtubules for sufficiently long times.

It is important to note that this section argues for the possibility of quantum coherence among microtubules within a single neuron. However, the manner in which this intracellular coherent entity integrates (or binds) with analogous coherent entities emerging in other neurons to give rise to a macro-experience remains to be explained. A potential solution to this binding problem may be found by considering the concept of quantum entanglement.

Entanglement is a kind of quantum correlation that binds spatially separated entities into a single, indivisible whole [67]. Although entanglement is fundamentally a quantum-mechanical phenomenon, it is not confined solely to subatomic particles or restricted to regimes of extremely low temperatures and short distances. Lee et al. [68] have demonstrated the creation of entanglement between 2-mm-sized oscillators at room temperature, separated by a distance of about 15 cm. In principle, entanglement can be created in a biomolecular system at physiologic temperatures if biomolecules oscillate and the system is pushed to a far-from-equilibrium state [69].

It has been demonstrated that entanglement can be generated between two separate, unlinked oscillators if each of them is properly coupled to a common network. In this situation, the emergence of synchronization between the two unlinked oscillators witnesses the presence of quantum entanglement [70]. Synchronization and entanglement are collective phenomena typically studied as two separate processes in the classical and quantum realms, respectively. However, Witthaut et al. [71] have provided some evidence showing that there is a direct relationship between classical synchronization and quantum entanglement. Specifically, in certain classes of quantum many-body systems, a significant part of the emergence of entanglement can be attributed to a classical synchronization process.

In the brain, neuronal network topology allows for the zero lag synchronization of distant populations of neurons. Vicente et al. [72] have shown that, even in the presence of significant axonal conduction delays, distant neuronal populations can self-organize to produce oscillations with zero phase lag. In other words, two neuronal groups that are both reciprocally connected to a common third group can exhibit oscillatory coherence at zero phase lag. Based on all these evidence and arguments, it can be hypothesized that, within the brain, the coupling of spatially separated neurons to a common cortical or subcortical node may promote the emergence of synchronization and consequently entanglement among those neurons. This could serve as a mechanism that binds micro-conscious entities together to form a macro-experience.

## Spontaneous emergence of coherence

The spontaneous emergence of coherence refers to a particular class of phase transitions by which many incoherent elements suddenly join together and transform into a single wave-like entity that is characterized by quantum coherence. In this process, individual elements lose their separate identities and collectively form a unified whole known as a matter-wave. This matter-wave is a real physical entity that (like the waves in the ocean) contains energy, fills a region of space, and has a value at every point throughout that region.

Similar to a sound wave or a light wave, a matter-wave also has an amplitude and a wavelength. The intensity of a matter-wave (i.e., the amount of energy it carries) is directly proportional to the square of the amplitude [73, p. 22]. The kinetic energy of a matter-wave is inversely proportional to its wavelength: the shorter the wavelength, the greater the kinetic energy [73, p. 142].

A Bose–Einstein condensate is the most classical form of a matter-wave [74]. Bose–Einstein condensation is a thermodynamic phase transition that occurs when the temperature of a population of particles (for example, a gas of sodium atoms) decreases below a critical threshold. As a consequence, some of the particles spontaneously enter into a single quantum state and collectively form a condensate which behaves like a coherent, classical wave [75].

In Bose – Einstein condensation of atoms, temperature is the parameter that drives the phase transition. However, it is also possible to keep the temperature constant and trigger the phase transition with increasing the density of the particles. In this scenario, density, instead of temperature, serves as the control parameter. Demidov et al. [76] have demonstrated that Bose – Einstein condensation can occur in a population of magnons at room temperature if the density of the population increases above a critical threshold. Magnons are quantum-mechanical counterparts of spin waves, and spin waves are delocalized excitations of a magnetic substrate [77].

Spontaneous coherence is the essence of Bose–Einstein condensation. It is so essential that, in order to confirm the existence of a Bose–Einstein condensate, one must demonstrate two things: first, there exists quantum coherence, and second, this coherence is spontaneous, not driven by an external coherent source [75]. In experimental studies that involve Bose–Einstein condensation of particles, the detection of long-range correlation among the particles is regarded as a direct evidence of the emergence of macroscopic quantum coherence [78]. In fact, long-range correlation among the components of a system is inherently linked to the spontaneous emergence of coherence in that system.

In addition to Bose–Einstein condensation, superconductivity and superfluidity are other notable examples of phenomena that involve the spontaneous emergence of coherence as a thermodynamic phase transition. The onset of coherent light emission from a laser device is also an example of spontaneous coherence, although in this case the quantum coherence does not arise from a thermodynamic phase transition [75]. Despite the fact that each of these four phenomena involves a different substrate and a different mechanism, they all belong to the same category of phenomena because all of them depend on the spontaneous emergence of quantum coherence in a system. Another phenomenon that also belongs to this category, according to the QBIT theory, is consciousness. In this case, the substrate is microtubules, and the mechanism involves synchronized spin-based oscillations. Most probably, the control parameter triggering the phase transition is neither temperature nor density, but something else, for example, the rate at which electrical energy is pumped into the microtubules, or the intensity of pumping. It is noteworthy that all synaptic inputs to the soma and dendrites of a neuron are combined and conveyed toward the axon hillock in the form of electrical energy. The funneling of this energy into the AIS by the rapid narrowing of the axon hillock may, in certain conditions, allow the control parameter to go beyond the critical threshold, hence triggering the phase transition in microtubules.

Therefore, a conjecture of the QBIT theory is that pumping of electrical energy into the AIS stimulates spintronic oscillations in microtubules and, under specific conditions, triggers the spontaneous emergence of spintronic coherence in microtubules. This idea is clearly inspired by the Fröhlich theory, but it differs from that theory in some details. According to Fröhlich [79], a protein can be considered as an array of coupled vibrational oscillators that is constantly stimulated by a supply of thermal energy. If the rate at which energy is pumped into the protein exceeds a critical threshold, then, through a process similar to Bose–Einstein condensation, the protein enters a state possessing quantum coherence. The Fröhlich theory is strongly supported by theoretical as well as experimental evidence [80–82]. Zhang et al. [83] have developed a full quantum statistical theory for the Fröhlich condensation, demonstrating that this phenomenon can lead to long-lived quantum coherence in proteins such as bovine serum albumin and lysozyme. Their findings show that the Fröhlich condensate is more like a laser rather than a Bose–Einstein condensation, although there is a striking similarity between these two phenomena.

It has been demonstrated that a phase transition similar to the Bose–Einstein condensation can actually occur at room temperature in a population of interacting spintronic oscillators, provided that the electrical current supplied to the group is sufficiently high and the distance separating the oscillators is adequately small [84,85]. These two conditions (i.e., a sufficiently large electrical current, and a sufficiently small distance between oscillators) are reminiscent of the situation of microtubules in the AIS, where they are closely packed together in fascicles and may be subjected to large electric currents pumped into the AIS form the axon hillock. Therefore, it could be argued that quantum coherence in microtubules, which seems to be unlikely to occur in the warm environment of the brain, might actually be feasible in the AIS due to its structural characteristics, strategic positioning, and the distinctive organization of microtubules. It is important to note that the spontaneous emergence of coherence in microtubules is consistently associated with consciousness, regardless of whether this occurs in the AIS, the soma, or the dendrites. However, according to the QBIT theory, the AIS is the only neuronal compartment where spontaneous coherence of microtubules is feasible.

When quantum coherence spontaneously emerges in a population of microtubules, all the microtubules join together (through long-range entanglement) and collectively form a unified whole: a matter-wave. Depending on the number of microtubules involved and the pattern of long-range entanglement among the microtubules, matter-waves with different characteristics are created. Each of these configurations, according to the QBIT theory, corresponds to a micro-consciousness (a quale). Simultaneous generation of multiple qualia by different parts of the cerebral cortex gives rise to the appearance of a macro-consciousness, the seemingly unified subjective experience that an individual has at any given moment.

## Warm, wet, and noisy

Historically, numerous proposals regarding the role of quantum phenomena in brain functions were dismissed based on the assertion that these phenomena are fragile and cannot be observed in the wet, warm, and noisy environments typical of biological systems. However, there is a growing body of evidence indicating that the famous “warm, wet, and noisy” argument is no longer valid. In this regard, the strongest and most convincing evidence comes from research on photosynthesis.

Photosynthesis depends on quantum coherence among chromophore molecules [86]. Hildner et al. [87] have demonstrated that, under physiological conditions, quantum coherence can persist at least 400 femtoseconds in the light harvesting protein (the LH2 complex) of a purple bacterium. This long-lived quantum coherence allows ultrafast energy transfer within individual LH2 complexes, which is a prerequisite for efficient light harvesting. Similarly, Lee et al. [88] have demonstrated that, in purple bacteria, long-lived quantum coherence is responsible for highly efficient energy harvesting and trapping during photosynthesis. A noteworthy aspect of this latter study is its indication that the long-range correlation present in the protein environment where photosynthesis takes place is the key factor that allows for the preservation of quantum coherence for sufficiently long times. Sarovar et al. [89] have demonstrated that, in the Fenna–Matthews–Olson (FMO) protein of the green sulfur bacteria, long-range and multipartite entanglement can exist for relatively long times even at physiological temperatures. In sum, there is now strong experimental evidence that photosynthetic organisms actually exploit quantum entanglement and coherence in real physiological conditions [90].

In general, in the principles of quantum mechanics, nothing (neither mass nor temperature) prevents macroscopic systems from exhibiting quantum phenomena [91]. For instance, Lee et al. [68] demonstrated quantum entanglement at room temperature between vibrational states of 2-mm-sized diamonds separated by a distance of about 15 cm. Riedinger et al. [92] created entanglement between the vibrations of two 10-μm-long silicon beams that were spatially separated by 20 cm.

## QBIT versus Orch OR

Orchestrated objective reduction (Orch OR) is a theoretical framework that explains consciousness in terms of quantum coherence and spin-based computations in microtubules [93]. Although the Orch OR and QBIT theories are largely consistent and compatible, a number of differences exist between the two theories. According to the Orch OR theory, consciousness emerges in dendrites and soma, specifically in their short and mixed polarity bundles of microtubules [94, p. 393]. By contrast, the QBIT theory suggests that it is the AIS (with its long, uniformly polarized microtubules) that serves as the strategic site for the generation of consciousness.

Another point of divergence between Orch OR and QBIT theories relate to the issue of protoconsciousness. The Orch OR theory maintains that a rudimentary form of consciousness (called protoconsciousness) is an intrinsic property of the universe [95]. This means that non-living entities, and even fundamental particles, have some rudimentary form of consciousness. However, the full-blown consciousness that humans and some animals possess depends on the evolution of biological structures like microtubules. In contrast to the Orch OR theory, the QBIT theory states that all forms of consciousness, including the most basic and rudimentary ones, are dependent upon microtubules. Consequently, according to this framework, no form of consciousness – however, primitive – could have existed in the universe prior to the emergence of life, and even the simplest forms of consciousness cannot be detected in any entity within the universe that lacks microtubules.

The Orch OR theory suggests that every objective reduction (OR) event is a protoconscious event [93]. In other words, each OR event is associated with a primitive experiential quality, no matter whether the OR occurs in the microtubules of a brain, in the molecules of a rock, or in the atoms of a gas. Since OR events are ubiquitous throughout the universe, protoconsciousness is also ubiquitous in the universe. This perspective is commonly referred to as panprotopsychism. Unlike the Orch OR theory, the QBIT theory does not lead to panprotopsychism. The QBIT theory suggests that consciousness necessarily requires the spontaneous emergence of coherence in a population of microtubules. While quantum coherence can and does occur in many different materials throughout the universe at any given moment, only spontaneous quantum coherence within microtubules – rather than in other substrates – is associated with the emergence of consciousness. In other words, although quantum coherence is a necessary condition for consciousness, its occurrence alone is not sufficient to produce conscious experience. Whenever there is consciousness, there is quantum coherence, but not vice versa: Quantum coherence is not identical to consciousness.

Before closing this section, it is important to emphasize that the QBIT theory does not constitute a fundamental theory in the same manner as Orch OR. At its current stage of development, the QBIT theory encompasses a considerably narrower scope compared to Orch OR. Specifically, the QBIT theory primarily addresses consciousness within the brain, whereas Orch OR proposes a broader framework concerning consciousness in the universe.

## The function of consciousness

In the same sense that electrical energy is a resource, quantum coherence is also a real physical resource that can be consumed by a system to perform a variety of tasks, including information processing and computational operations [96,97]. Quantum coherence not only improves the efficiency of information processing beyond what is achievable by classical resources, but it also enables computations that are fundamentally impossible to perform with classical resources like electrical energy [98].

According to the QBIT theory, whenever there is consciousness, there is quantum coherence. In a broad sense, consciousness is nothing but quantum coherence in a population of brain microtubules. Therefore, since quantum coherence is a resource, consciousness may similarly be viewed as a resource. Based on the resource theory of coherence, two primary functions can be attributed to consciousness: first, consciousness enhances the efficiency of information processing within the brain, and second, consciousness enables the brain to perform computations that are absolutely impossible without consciousness.

## Falsifiability of the QBIT theory

Matter-wave interferometry is an experimental setup that has been successfully used to reveal the wave nature of several biomolecules such as tetraphenylporphyrin [99] and gramicidin [100]. One proposition of the QBIT theory is that, under certain circumstances, microtubules transform into a matter-wave. An experiment could be designed to investigate whether the proposed wave nature of microtubules can be observed using a matter-wave interferometer. The QBIT theory will be falsified if such experiments fail to show the wave nature of microtubules. On the other hand, the observation of de Broglie wave interference in microtubules will provide some experimental support for the QBIT theory, although this does not constitute a definitive proof of the theory.

The QBIT theory will be invalidated if experiments demonstrate that the phenomenon known as spontaneous coherence is fundamentally impossible in microtubules at room temperature. Furthermore, the theory will be undermined to a great extent if experiments fail to show any spintronic properties in microtubules. An initial step in assessing the potential spintronic properties of microtubules involves investigating whether tubulin exhibits the chiral-induced spin selectivity (CISS) effect. This can be explored through a range of experimental techniques, including photoelectron spectroscopy, spin-polarized conductive atomic force microscopy, and Hall voltage measurements. To date, the CISS effect has been unequivocally demonstrated in several proteins and peptides, such as bacteriorhodopsin and polyalanine [101]; however, its presence in tubulins has yet to be investigated.

## The explanatory gap

The main challenge confronting all theories of consciousness is bridging the explanatory gap. In fact, no theory can be deemed complete unless it provides a plausible account that addresses this gap. Since Levine [102] first introduced the concept of the “explanatory gap,” numerous efforts have been undertaken to bridge this divide; however, none have achieved widespread consensus or definitive success. The QBIT theory does not offer a more compelling solution to the explanatory gap compared to other theories of consciousness. In fact, the central weakness of the QBIT theory lies in its failure to provide a robust and convincing account addressing the gap. However, the argument presented in this section – albeit tentative and preliminary – may be viewed as a rudimentary attempt to engage with this foundational issue.

According to the QBIT theory, the spontaneous emergence of quantum coherence within a population of microtubules leads to the formation of a new physical entity, referred to as a matter-wave, which is fundamentally a collective entity derived from microtubules. The theory then suggests that this specific microtubule-based collective entity is responsible for the existence of subjective experience in our universe. Spontaneous coherence can manifest in various other physical entities, such as sodium atoms, resulting in the creation of a collective entity (matter-wave) derived from sodium atoms. This raises a question: why is a matter-wave originating from microtubules associated with consciousness, while a matter-wave from sodium atoms is not? On one side, we have a physical entity, and on the other, we have a characteristic of that entity; our goal is to clarify why this physical entity possesses that characteristic, while another similar physical entity does not.

A parallel can be drawn from other domains of science. For example, the Higgs field is a quantum field responsible for giving mass to objects. The electromagnetic field is also a quantum field but lacks the ability to confer mass; instead, it can illuminate a dark room. Although both are classified as quantum fields, why the Higgs field can give mass to objects, while the electromagnetic field cannot? Why is it that the electromagnetic field can brighten a dark room, yet the Higgs field cannot? Is there not an explanatory gap between a physical entity and its properties in this context? We do not perceive a gap here and simply accept that the ability to give mass to objects is an intrinsic property of the Higgs field. Just as we accept and are at ease with the idea that the Higgs field possesses certain intrinsic properties that cannot be replicated by other fields, why do we find it challenging to accept that being responsible for consciousness is merely an intrinsic characteristic of the microtubule matter-wave?

The argument put forth in this section does not aim to close the explanatory gap nor does it deny the existence of such a gap initially. Rather, it aims to raise the possibility that we might be overly sensitive to the explanatory gap in consciousness studies, while a similar variant of this gap persists in other domains of science without disturbing us. In certain scientific fields, we experience less discomfort and unease in accepting that a weird characteristic (X) is an inherent property of Y, without pressing for an explanation as to why X is solely the intrinsic trait of Y and not of Z or W. In the same sense that the Higgs field is responsible for the mass in the universe, one can argue that a collective entity (a matter-field) derived from microtubules is responsible for phenomenal consciousness in the universe. The only difference between these two examples lies in the fact that there is a consensus that mass is objective but there is not a consensus that phenomenal consciousness is objective; rather, it seems to be something very special and subjective. This invites reflection on whether we are excessively sensitive to an explanatory gap in the context of consciousness.

## Final remarks

Microtubules are involved not only in the emergence of consciousness but also in the encoding and storage of memory within the brain. Evidence suggests that microtubules have a large potential for memory storage through phosphorylation mediated by Ca^2+^-calmodulin dependent kinase II (CaMKII), and that the memory thus encoded on microtubules is able to regulate membrane potential and synaptic plasticity [103]. It is noteworthy that tau, a microtubule-associated protein primarily localized in axons and known for stabilizing microtubules against depolymerization, also plays a significant role in memory and synaptic plasticity [104].

The involvement of microtubules in consciousness and memory is closely linked to the coordinated functioning of other cytoskeletal elements, including actin filaments and intermediate filaments. It has been proposed that actin filaments within synaptic spines facilitate the transmission of postsynaptic electrical signals, conveyed as ion waves, toward the microtubule network. Within this network, these signals are subsequently processed and utilized to regulate synaptic plasticity [105]. Consequently, a critical function of actin filaments may lie in mediating the interaction between ion channels and membrane potential on the one hand and microtubules on the other hand.

The cortical cytoskeleton of axons consists of short actin filaments which are organized into ring-like structures wrapping around the circumference of the axon. Spectrin tetramers connect the neighboring actin rings along the long axis of the axon, which maintain a regular spacing of approximately 180 nm, thereby giving the axonal cytoskeleton a long-range order [106]. Although there are differences in molecular composition between the AIS and the distal axon, the overall cytoskeletal architecture remains similar in both regions, adopting a periodic and highly ordered pattern. Notably, this periodic arrangement of actin filaments is unique to axons and is not found in dendrites, which instead primarily contains long actin filaments running along the dendritic axis [106]. The long-range order of the cytoskeleton that is present in the AIS and distal axon (but absent in dendrites and soma) may further facilitate the emergence of coherent oscillations within the AIS, a phenomenon suggested by the QBIT theory as essential for the emergence of consciousness.

Since the concept of matter-field is at the core of the QBIT theory, the conjectures of this theory can be better understood through the perspective of field theories of consciousness. Among the different field theories of consciousness, an interesting new theory, the general resonance theory [107], deserves special attention here.

Similar to the QBIT theory, the general resonance theory (GRT) suggests that different neuronal populations generate micro-conscious entities that should be combined together to form a macro-consciousness [108]. While the QBIT theory assumes that quantum entanglement is responsible for the binding of distributed micro-conscious entities into a unified macro-consciousness, the GRT suggests that it is the shared resonance among neuronal assemblies that causes micro-conscious entities to integrate into macro-conscious entities [107]. This resonance often induces a phase transition in the speed of information sharing, which enables the emergence of more complex forms of consciousness. According to the GRT, consciousness starts as a rudimentary form and becomes more complex and enriched through a resonance-based mechanism. Within the framework of GRT, resonance is conceptually analogous to field coherence; however, this form of coherence should not be conflated with quantum coherence, which the QBIT theory identifies as a necessary condition for consciousness. Therefore, unlike the QBIT theory, the GRT does not require to argue for the possibility of quantum phenomena in the warm and noisy environment of the brain to support its theoretical validity.

## Data Availability

This paper has no associated data.

## Declarations

Declaration of generative AI and AI-assisted technologies in the writing process: The author did not used AI and AI-assisted technologies in the writing process.
